# Supplementary material for: The Association of Redox Regulatory Drug Target Genes with Psychiatric Disorders: A Mendelian Randomization Study
Source: Antioxidants (Basel). 2024 Mar 27;13(4):398. doi: 10.3390/antiox13040398 (PMC11047424; doi:10.3390/antiox13040398)
Supplement: Supplementary file 1 [file antioxidants-13-00398-s001.zip › Supplementary Materials 1 Supplementary TableS1-S10.pdf]

Table S1. Antioxidants target genes.

Antioxidant Drugs	Mechanism of action	ChEMBL	Targets	Drug Bank	Targets
N-Acetylcysteine (NAC)	Salvianolic acid B like resveratrol is an activator of SIRT1 and an inhibitor of NF-κB	NA	*NA*	Glutathione synthetase, Cystine/glutamate transporter, Aminoacylase-1, Inhibitor of nuclear factor kappa-B kinase subunit beta, Inhibitor of nuclear factor kappa-B kinase subunit alpha, Glutamate receptor ionotropic, NMDA 1/2A/2B/2D/3A	*GSS* (stimulator), *SLC7A11* (activator), *ACY1* (substrate), *IKBKB* (inhibitor), *CHUK* (inhibitor), *GRIN1* (activator), *GRIN2A* (activator), *GRIN2B* (activator), *GRIN2D* (activator), *GRIN3A* (activator)
Ginkgo biloba extract	Ginko biloba extract<br>(i) Scavenger of superoxide anion, hydroxyl radicals, peroxyl<br>(ii) Reduces the concentration of primary and secondary products of lipid peroxidation (diene conjugates, TBA-active products)<br>(iii) Induces the enzymatic activity of superoxide dismutase and catalase	NA	*NA*	Sodium-dependent noradrenaline transporter, Phospholipase A2 membrane associated, Glycine receptor subunit alpha-1, Gamma-aminobutyric acid receptor subunit alpha-1/beta-2/gamma-2, Amine oxidase [flavin-containing] A, Nitric oxide synthase, inducible	*SLC6A2* (inhibitor), *PLA2G2A* (inhibitor), *GLRA1* (antagonist), *GABRA1* (negative modulator), *GABRB2* (negative modulator), *GABRG2* (negative modulator), *MAOA* (inhibitor), *NOS2* (inhibitor)
Selegiline	The antioxidant effect of selegiline may be associated with the protection of neurons from oxygen free radicals that are released as a result of MAO-B activity	Monoamine oxidase B	*MAOB* (inhibitor)	Amine oxidase [flavin-containing] B, Amine oxidase [flavin-containing] A	*MAOA* (inhibitor), *MAOB* (inhibitor)
Allopurinol	Allopurinol prevents the formation of free radicals:<br>(i) Inhibits the xanthine oxidase reaction, during which superoxide anion radical is actively formed	Xanthine dehydrogenase	*XDH* (inhibitor)	Xanthine dehydrogenase/oxidase	*XDH* (inhibitor)
Vitamin E	Vitamin E is a natural antioxidant that neutralizes free radicals:<br>(i) There is a transfer of hydrogen of the phenyl group to the peroxide radical<br>(ii) Performs structural function by interacting with phospholipids of biological membranes<br>(iii) Restores the oxidation product of tocopherol (α-tocopheroxide) to α-tocopherol	NA	*NA*	SEC14-like protein 4/3/2, Nuclear receptor subfamily 1 group I member 2, Protein kinase C beta/alpha type, Arachidonate 5-lipoxygenase, Diacylglycerol kinase alpha, Serine/threonine-protein phosphatase 2A catalytic subunit beta/alpha isoform	*SEC14L2* (NA), *SEC14L3* (NA), *SEC14L4* (NA), *NR1I2* (NA), *PRKCB* (NA), *PRKCA* (NA), *ALOX5* (NA), *DGKA* (NA), *PPP2CB* (NA), *PPP2CA* (NA)
Vitamin C	Vitamin C antioxidant activity:<br>(i) The formation of a redox pair of ascorbic acid/dehydroascorbic acid,<br>restores the active form of vitamin E<br>(ii) Prevents or reverses the oxidation process of reduced glutathione (GSH) to its functionally inactive form (GSSG)	NA	*NA*	Procollagen-lysine,2-oxoglutarate 5-dioxygenase 2/3, Phytanoyl-CoA dioxygenase peroxisomal, Gamma-butyrobetaine dioxygenase, Dopamine beta-hydroxylase, Peptidyl-glycine alpha-amidating monooxygenase, Prolyl 3-hydroxylase 1/2/3, Prolyl 4-hydroxylase subunit alpha-1, 2-oxoglutarate and iron-dependent oxygenase domain-containing protein 1/2, Alpha-ketoglutarate-dependent dioxygenase alkB homolog 2/3, Lysine-specific demethylase 5D, Procollagen-lysine,2-oxoglutarate 5-dioxygenase 1, Trimethyllysine dioxygenase, mitochondrial, Transmembrane prolyl 4-hydroxylase, Egl nine homolog 1/2/3	*PLOD2* (cofactor), *PLOD3* (cofactor), *PHYH* (cofactor), *BBOX1* (cofactor), *DBH* (cofactor), *PAM* (cofactor), *P3H1* (cofactor), *P3H2* (cofactor), *P3H3* (cofactor), *P4HA1* (cofactor), *OGFOD1* (cofactor), *OGFOD2* (cofactor), *ALKBH2* (activator), *ALKBH3* (activator), *KDM5D* (cofactor), *PLOD1* (cofactor), *TMLHE* (cofactor), *P4HTM* (cofactor), *EGLN1* (cofactor), *EGLN2* (cofactor), *EGLN3* (cofactor)
Polyunsaturated fatty acids (PUFAs)/Omega-3	The detailed mechanisms of action are mostly unknown, but PUFAs have anti-inflammatory and antiatherogenic effects	NA	*NA*	Peroxisome proliferator-activated receptor gamma, Peroxisome proliferator-activated receptor alpha, Sterol regulatory element-binding protein 1	*PPARG* (ligand), *PPARA* (activator), *SREBF1* (inhibitor)
Curcumin	Inhibit Keap1 protein through electrophilic modification, which leads to Nrf2 activation	NA	*NA*	Peroxisome proliferator-activated receptor gamma, Vitamin D3 receptor, Multidrug resistance-associated protein 5, Carbonyl reductase [NADPH] 1, Glutathione S-transferase P	*PPARG* (NA), *VDR* (NA), *ABCC5* (inhibitor), *CBR1* (NA), *GSTP1* (NA)
Resveratrol	Resveratrol is<br>(i) An activator of NAD-dependent deacetylase sirtuin-1 (SIRT1), which<br>activates the transcription factor FoxO<br>(ii) An inhibitor of NF-κB (anti-inflammatory effect)	NA	*NA*	Ribosyldihydronicotinamide dehydrogenase [quinone], Casein kinase II subunit alpha, Prostaglandin G/H synthase 1/2, Arachidonate 15-lipoxygenase, Arachidonate 5-lipoxygenase, Aryl hydrocarbon receptor, Phosphatidylinositol 4-kinase type 2-beta, Integrin alpha-5/beta-3, Amyloid beta A4 protein, Alpha-synuclein, NAD-dependent protein deacetylase sirtuin-1, Estrogen receptor alpha, Melatonin receptor type 1A/1B, C-type lectin domain family 14 member A, Nuclear receptor subfamily 1 group I member 2/3, Solute carrier family 2, facilitated glucose transporter member 1, Carbonyl reductase [NADPH] 1, Peroxisome proliferator-activated receptor alpha/gamma, RAC-alpha serine/threonine-protein kinase, Far upstream element-binding protein 2, Tyrosine--tRNA ligase, cytoplasmic	*NQO2* (NA), *CSNK2A2* (NA), *PTGS2* (inhibitor), *PTGS1* (inhibitor), *ALOX15* (NA), *ALOX5* (NA), *AHR* (NA), *PI4K2B* (NA), *ITGA5* (NA), *ITGB3* (NA), *APP* (NA), *SNCA* (NA), *SIRT1* (NA), *ESR1* (NA), *MTNR1A* (NA), *MTNR1B* (NA), *CLEC14A* (NA), *NR1I2* (NA), *NR1I3* (NA), *SLC2A1* (NA), *CBR1* (inhibitor), *PPARG* (NA), *PPARA* (NA), *AKT1* (inhibitor), *FBP2* (NA), *YARS1* (inhibitor)
Metformin	Activate FoxO3 via AMP-activated protein kinase activation	Mitochondrial glycerol-3-phosphate dehydrogenase	*GPD2*	Electron transfer flavoprotein-ubiquinone oxidoreductase, mitochondrial, 5'-AMP-activated protein kinase subunit beta-1, Glycerol-3-phosphate dehydrogenase [NAD(+)], cytoplasmic	*ETFDH* (inhibitor), *PRKAB1* (inducer activator), *GPD1* (inhibitor)
Vitamin D		Vitamin D receptor	*VDR*	Vitamin D3 receptor, Vitamin D-binding protein	*VDR* (NA), *GC* (NA)
Vitamin A		NA	*NA*	Apolipoprotein D, Prostaglandin-H2 D-isomerase, Retinol dehydrogenase 12, 11-cis retinol dehydrogenase, Retinol dehydrogenase 13/1/2, Lecithin retinol acyltransferase, Short-chain dehydrogenase/reductase 3, Aldehyde dehydrogenase family 1 member A3, All-trans-retinol 13,14-reductase, Retinol dehydrogenase 11, Dehydrogenase/reductase SDR family member 4, Retinol dehydrogenase 14, Retinol dehydrogenase 8	*APOD* (ligand), *PTGDS* (ligand), *RDH5* (substrate), *RDH12* (substrate), *ALDH1A2* (substrate), *ALDH1A1* (substrate), *LCAT* (substrate), *DHRS3* (substrate), *ALDH1A3* (substrate), *RETSAT* (substrate), *RDH11* (substrate), *DHRS4* (substrate), *RDH8* (substrate)
coenzyme Q/ubiquinone		NA	*NA*	NADH dehydrogenase [ubiquinone] flavoprotein 3, mitochondrial, Succinate dehydrogenase [ubiquinone] flavoprotein subunit, mitochondrial	*NDUFV3* (cofactor), *SDHA* (cofactor)
Melatonin		Melatonin receptor	*MTNR1B*, *MTNR1A*	Melatonin receptor 1A, Melatonin receptor 1B, Estrogen receptor alpha, Nuclear receptor ROR-beta, Calmodulin, Myeloperoxidase, Eosinophil peroxidase, Calreticulin, Acetylserotonin O-methyltransferase, Ribosyldihydronicotinamide dehydrogenase [quinone]	*MTNR1B* (agonist), *MTNR1A* (agonist), *ESR1* (antagonist), *RORB* (agonist), *CALM1* (NA), *MPO* (inhibitor), *EPX* (inhibitor), *CALR* (NA), *ASMT* (NA), *NQO2* (NA)
Piracetam		NA	*NA*	NA	*NA*
Sulforaphane	Inhibit Keap1 protein through electrophilic modification, which leads to Nrf2 activation. The transcription factor Nrf2 plays a central role in the inducible expressions of many cytoprotective genes in response to oxidative stress	NA	*NA*	NA	*NA*
DDO-7263	Nrf2 activation and NLRP3 inflammasome inhibition	NA	*NA*	NA	*NA*
Salvianolic acid B	Salvianolic acid B like resveratrol is an activator of SIRT1 and an inhibitor of NF-κB	NA	*NA*	NA	*NA*

NA: not applicable

Table S2. Data sources in this study.				
**Exposures**	**PMID/URL**	**Ancestry**	**Dataset or consortium**	**Participants**
**eQTL**				
Brain - Amygdala	32913098	Predominantly European (~85%)	GTEx V8	152
Brain - Anterior cingulate cortex (BA24)				176
Brain - Caudate (basal ganglia)				246
Brain - Cerebellar Hemisphere				215
Brain - Cerebellum				241
Brain - Cortex				255
Brain - Frontal Cortex (BA9)				209
Brain - Hippocampus				197
Brain - Hypothalamus				202
Brain - Nucleus accumbens (basal ganglia)				246
Brain - Putamen (basal ganglia)				205
Brain - Spinal cord (cervical c-1)				159
Brain - Substantia nigra				139
**pQTL**				
Dorsolateral prefrontal cortex	29865057	European	ROSMAP	376
**Outcomes**				
Schizophrenia	35396580	European	PGC	53,386 cases and 77,258 controls
Bipolar disorder	34002096	European	PGC	41,917 cases and 371,549 controls
Major depressive disorder	30718901	European	PGC	170,756 cases and 329,443 controls
Obsessive-compulsive disorder	28761083	European	PGC	2688 cases and 7037 controls
Attention-deficit/hyperactivity disorder	30478444	European	PGC	20,183 cases and 35,191 controls
Autism spectrum disorder	30804558	European	PGC	18,381 cases and 27,969 controls
Anorexia nervosa	31308545	European	PGC	16,992 cases and 55,525 controls
**Mediators**				
Vitamin C	https://gwas.mrcieu.ac.uk/datasets/ukb-b-19390/	European	UK biobank	64,979
Vitamin E	https://gwas.mrcieu.ac.uk/datasets/ukb-b-6888/	European	UK biobank	64,979
Fluid intelligence				
Fluid intelligence score	https://gwas.mrcieu.ac.uk/datasets/ukb-b-5238/	European	UK biobank	149,051
Trail marking				
Duration to complete numeric path	https://gwas.mrcieu.ac.uk/datasets/ukb-b-16026/	European	UK biobank	99,479
Duration to complete alphanumeric path	https://gwas.mrcieu.ac.uk/datasets/ukb-b-20140/	European	UK biobank	99,477
Pairs matching				
Number of incorrect matches in round	https://gwas.mrcieu.ac.uk/datasets/ukb-b-20498/	European	UK biobank	462,302
Number of correct matches in round	https://gwas.mrcieu.ac.uk/datasets/ukb-b-2084/	European	UK biobank	462,302
Numeric Memory				
Maximum digits remembered correctly	https://gwas.mrcieu.ac.uk/datasets/ukb-b-18683/	European	UK biobank	106,162
Prospective Memory				
Prospective memory result	https://gwas.mrcieu.ac.uk/datasets/ukb-b-4282/	European	UK biobank	152,605

Expression quantitative trait loci, eQTL; protein quantitative trait loci, pQTL; Genotype-Tissue Expression, GTEx, version 8; Religious Orders Study and Rush Memory and Aging Project, ROSMAP; Psychiatric Genomics Consortium, PGC.

Table S3. Two-sample MR of the targets on schizophrenia.

	SNPs	Test	SNP_number	Beta	Se	*P_value*	Genes
**NAC**							
**Amygdala**							
	rs11644783	Wald ratio	1	0.135	0.044	2.14E-03	*GRIN2A*
**Cerebellar_Hemisphere**							
	rs12003467	Wald ratio	1	0.045	0.020	2.67E-02	*GRIN3A*
**Cerebellum**							
	rs6058209	Wald ratio	1	-0.126	0.039	1.20E-03	*GSS*
**Cortex**							
	rs1060615	Wald ratio	1	-0.139	0.056	1.34E-02	*GSS*
**Spinal_cord_cervical**							
	rs76529537	Wald ratio	1	0.146	0.059	1.35E-02	*IKBKB*
**Substantia_nigra**							
	rs3105688	Wald ratio	1	0.037	0.013	3.27E-03	*GRIN2A*
**ROSMAP**							
	rs2236271	Wald ratio	1	-0.381	0.159	1.67E-02	*GSS*
	rs6533014, rs1598856	Inverse variance weighted	2	-0.824	0.260	1.55E-03	*NFKB1*
**Ubiquinone**							
**Amygdala**							
	rs2839606	Wald ratio	1	0.071	0.028	1.26E-02	*NDUFV3*
**Anterior_cingulate_cortex**							
	rs11203218	Wald ratio	1	0.072	0.030	1.54E-02	*NDUFV3*
**Putamen_basal_ganglia**							
	rs2839606	Wald ratio	1	0.060	0.024	1.26E-02	*NDUFV3*
**Vitamin C**							
**Amygdala**							
	rs111899460	Wald ratio	1	0.074	0.033	2.29E-02	*P3H2*
**Anterior_cingulate_cortex**							
	rs10865952	Wald ratio	1	0.242	0.067	2.80E-04	*P4HTM*
**Cerebellum**							
	rs28569885	Wald ratio	1	-0.244	0.047	2.14E-07	*OGFOD2*
**Frontal_Cortex**							
	rs13064784	Wald ratio	1	-0.153	0.046	9.65E-04	*P4HTM*
	rs28768122	Wald ratio	1	-0.204	0.032	1.15E-10	*OGFOD2*
	rs6571602	Wald ratio	1	0.204	0.078	9.31E-03	*EGLN3*
**Hypothalamus**							
	rs6446205	Wald ratio	1	0.151	0.047	1.43E-03	*P4HTM*
**Nucleus_accumbens**							
	rs2739514	Wald ratio	1	-0.085	0.042	4.43E-02	*EGLN1*
**Putamen_basal_ganglia**							
	rs2002556	Wald ratio	1	0.109	0.045	1.51E-02	*PLOD1*
**Spinal_cord_cervical**							
	rs6791234	Wald ratio	1	0.063	0.020	1.60E-03	*P4HTM*
**ROSMAP**							
	rs1449453	Wald ratio	1	0.272	0.110	1.32E-02	*PLOD2*
**Vitamin E**							
**Amygdala**							
	rs9895770	Wald ratio	1	-0.089	0.044	4.34E-02	*PRKCA*
**Cerebellar_Hemisphere**							
	rs5753113	Wald ratio	1	0.135	0.038	4.40E-04	*SEC14L2*
**Cerebellum**							
	rs35289401	Wald ratio	1	0.096	0.045	3.41E-02	*SEC14L2*
**Omega-3**							
**Anterior_cingulate_cortex**							
	rs12600694	Wald ratio	1	-0.160	0.053	2.54E-03	*SREBF1*
**Spinal_cord_cervical**							
	rs116527582	Wald ratio	1	0.084	0.039	3.11E-02	*PPARG*
**Vitamin A**							
**Anterior_cingulate_cortex**							
	rs28691263	Wald ratio	1	0.066	0.031	3.19E-02	*APOD*
	rs908304	Wald ratio	1	-0.145	0.042	5.63E-04	*RETSAT*
**ROSMAP**							
	rs7179609, rs493258, rs17269397	Inverse variance weighted	3	0.077	0.035	2.95E-02	*ALDH1A2*
**Metformin**							
**Caudate_basal_ganglia**							
	rs1978108	Wald ratio	1	-0.130	0.038	6.38E-04	*PRKAB1*
**Cerebellar_Hemisphere**							
	rs8178290	Wald ratio	1	0.074	0.033	2.33E-02	*EPX*
**Cerebellum**							
	rs7310266	Wald ratio	1	-0.144	0.042	5.46E-04	*PRKAB1*
	rs836178	Wald ratio	1	-0.119	0.036	8.03E-04	*GPD1*
**Putamen_basal_ganglia**							
	rs7304996	Wald ratio	1	-0.113	0.033	5.89E-04	*PRKAB1*
**ROSMAP**							
	rs278155	Wald ratio	1	1.621	0.527	2.11E-03	*PRKAB1*
	rs13425727	Wald ratio	1	-0.981	0.377	9.27E-03	*GPD2*
**Melatonin**							
**Caudate_basal_ganglia**							
	rs8177477	Wald ratio	1	-0.146	0.047	2.13E-03	*CALR*
**Cerebellum**							
	rs514209	Wald ratio	1	0.234	0.041	8.80E-09	*RORB*
**Hypothalamus**							
	rs148781627	Wald ratio	1	-0.051	0.020	1.04E-02	*RORB*
	rs3863211	Wald ratio	1	-0.062	0.020	1.51E-03	*NQO2*
**Putamen_basal_ganglia**							
	rs4744709	Wald ratio	1	0.198	0.069	4.40E-03	*RORB*
**Spinal_cord_cervical**							
	rs62082141	Wald ratio	1	-0.055	0.019	4.24E-03	*MPO*
**ROSMAP**							
	rs318489, rs17136117, rs17300141	Inverse variance weighted	3	-0.084	0.038	2.58E-02	*NQO2*
**Ginkgo biloba extract**							
**Cerebellar_Hemisphere**							
	rs55689355	Wald ratio	1	-0.053	0.015	4.07E-04	*GLRA1*
**Cerebellum**							
	rs56392306	Wald ratio	1	-0.050	0.017	2.40E-03	*GLRA1*
**Resveratrol**							
**Hippocampus**							
	rs4817078	Wald ratio	1	-0.159	0.050	1.61E-03	*APP*
**Hypothalamus**							
	rs3863211	Wald ratio	1	-0.062	0.020	1.51E-03	*NQO2*
**Nucleus_accumbens**							
	rs4697589	Wald ratio	1	-0.075	0.036	3.95E-02	*PI4K2B*
**Spinal_cord_cervical**							
	rs116527582	Wald ratio	1	0.084	0.039	3.11E-02	*PPARG*
**ROSMAP**							
	rs318489, rs17136117, rs17300141	Inverse variance weighted	3	-0.084	0.038	2.58E-02	*NQO2*
**Curcumin**							
**Spinal_cord_cervical**							
	rs116527582	Wald ratio	1	0.084	0.039	3.11E-02	*PPARG*
**ROSMAP**							
	rs7621975	Wald ratio	1	0.775	0.333	1.98E-02	*ABCC5*

NAC, N-Acetylcysteine

ROSMAP, Religious Orders Study and Rush Memory and Aging Project

Table S4. Two-sample MR of the targets on bipolar disorder.							
	SNPs	Test	SNP_number	Beta	Se	*P_value*	Genes
**NAC**							
**Amygdala**							
	rs3751972	Wald ratio	1	0.073	0.023	1.92E-03	*NOS2*
**Caudate_basal_ganglia**							
	rs12932206	Wald ratio	1	0.076	0.036	3.44E-02	*GRIN2A*
**Cerebellar_Hemisphere**							
	rs146285957	Wald ratio	1	-0.228	0.109	3.56E-02	*GRIN1*
	rs12003467	Wald ratio	1	0.056	0.022	9.68E-03	*GRIN3A*
**Cerebellum**							
	rs6058209	Wald ratio	1	-0.083	0.042	4.76E-02	*GSS*
	rs1337695	Wald ratio	1	0.043	0.019	2.53E-02	*GRIN3A*
**Cortex**							
	rs72765195	Wald ratio	1	0.203	0.103	4.94E-02	*GRIN1*
**Frontal_Cortex**							
	rs6479037	Wald ratio	1	-0.197	0.080	1.42E-02	*GRIN3A*
**Hippocampus**							
	rs12932206	Wald ratio	1	0.114	0.054	3.44E-02	*GRIN2A*
**Putamen_basal_ganglia**							
	rs12932206	Wald ratio	1	0.055	0.026	3.44E-02	*GRIN2A*
**Spinal_cord_cervical**							
	rs12932206	Wald ratio	1	0.032	0.015	3.44E-02	*GRIN2A*
**Substantia_nigra**							
	rs57283800	Wald ratio	1	-0.263	0.076	5.39E-04	*GRIN1*
	rs3105688	Wald ratio	1	0.045	0.014	1.08E-03	*GRIN2A*
**ROSMAP**							
	rs6533014, rs1598856	Inverse variance	2	-0.595	0.290	4.05E-02	*NFKB1*
**Vitamin E**							
**Amygdala**							
	rs9895770	Wald ratio	1	-0.188	0.048	7.69E-05	*PRKCA*
**ROSMAP**							
	rs120908	Wald ratio	1	-1.395	0.248	1.24E-05	*PRKCB*
	rs8078231, rs741141, rs6504459, rs1995695	Inverse variance	4	-0.613	0.263	1.97E-02	*PRKCA*
**Ginkgo biloba extract**							
**Anterior_cingulate_cortex**							
	rs4796172	Wald ratio	1	-0.048	0.017	6.04E-03	*NOS2*
**Caudate_basal_ganglia**							
	rs3751972	Wald ratio	1	-0.038	0.012	1.92E-03	*NOS2*
**Cerebellar_Hemisphere**							
	rs55689355	Wald ratio	1	-0.043	0.016	8.46E-03	*GLRA1*
	rs3751972	Wald ratio	1	-0.042	0.014	1.92E-03	*NOS2*
**Cerebellum**							
	rs56392306	Wald ratio	1	-0.052	0.018	3.63E-03	*GLRA1*
	rs3751972	Wald ratio	1	-0.039	0.013	1.92E-03	*NOS2*
**Hippocampus**							
	rs11657699	Wald ratio	1	0.045	0.016	4.11E-03	*NOS2*
**Hypothalamus**							
	rs3751972	Wald ratio	1	0.089	0.029	1.92E-03	*NOS2*
**Nucleus_accumbens**							
	rs3751972	Wald ratio	1	-0.038	0.012	1.92E-03	*NOS2*
**Putamen_basal_ganglia**							
	rs3751972	Wald ratio	1	0.061	0.020	1.92E-03	*NOS2*
**Spinal_cord_cervical**							
	rs3751972	Wald ratio	1	0.062	0.020	1.92E-03	*NOS2*
**Substantia_nigra**							
	rs3751972	Wald ratio	1	0.067	0.022	1.92E-03	*NOS2*
**Vitamin A**							
**Anterior_cingulate_cortex**							
	rs908304	Wald ratio	1	-0.112	0.046	1.46E-02	*RETSAT*
**Cerebellar_Hemisphere**							
	rs7146599	Wald ratio	1	0.044	0.020	2.97E-02	*DHRS4*
**Cerebellum**							
	rs7146599	Wald ratio	1	0.044	0.020	2.97E-02	*DHRS4*
**Spinal_cord_cervical**							
	rs61775046	Wald ratio	1	-0.160	0.059	6.96E-03	*DHRS3*
**Resveratrol**							
**Anterior_cingulate_cortex**							
	rs7210182	Wald ratio	1	-0.092	0.027	5.47E-04	*ITGB3*
**Cortex**							
	rs147286120	Wald ratio	1	0.115	0.038	2.63E-03	*ITGB3*
	rs75756429	Wald ratio	1	0.077	0.032	1.78E-02	*ESR1*
**Frontal_Cortex**							
	rs4541130	Wald ratio	1	-0.086	0.042	3.84E-02	*ITGB3*
**Hypothalamus**							
	rs3863211	Wald ratio	1	-0.053	0.021	1.21E-02	*NQO2*
	rs530425	Wald ratio	1	-0.068	0.031	2.84E-02	*PPARG*
**Nucleus_accumbens**							
	rs12942340	Wald ratio	1	-0.066	0.033	4.78E-02	*ITGB3*
**Spinal_cord_cervical**							
	rs116527582	Wald ratio	1	0.114	0.042	6.18E-03	*PPARG*
**Melatonin**							
**Caudate_basal_ganglia**							
	rs8177477	Wald ratio	1	-0.104	0.049	3.56E-02	*CALR*
**Cerebellum**							
	rs514209	Wald ratio	1	0.095	0.044	3.10E-02	*RORB*
**Cortex**							
	rs75756429	Wald ratio	1	0.077	0.032	1.78E-02	*ESR1*
**Hypothalamus**							
	rs3863211	Wald ratio	1	-0.053	0.021	1.21E-02	*NQO2*
**Spinal_cord_cervical**							
	rs62082141	Wald ratio	1	-0.055	0.019	4.24E-03	*MPO*
**Substantia_nigra**							
	rs11668444	Wald ratio	1	0.075	0.036	3.92E-02	*CALR*
**Vitamin C**							
**Cerebellar_Hemisphere**							
	rs4660653	Wald ratio	1	0.107	0.052	3.72E-02	*P3H1*
	rs4963510	Wald ratio	1	0.108	0.050	2.96E-02	*P3H3*
	rs4384984	Wald ratio	1	0.096	0.041	1.98E-02	*P4HTM*
**Cerebellum**							
	rs115117128	Wald ratio	1	0.080	0.038	3.55E-02	*P3H1*
	rs4963510	Wald ratio	1	0.057	0.026	2.96E-02	*P3H3*
**Cortex**							
	rs4963510	Wald ratio	1	0.095	0.044	2.96E-02	*P3H3*
**Frontal_Cortex**							
	rs28768122	Wald ratio	1	-0.086	0.035	1.31E-02	*OGFOD2*
**Substantia_nigra**							
	rs147952742	Wald ratio	1	0.075	0.034	2.59E-02	*OGFOD2*
**Omega-3**							
**Hypothalamus**							
	rs530425	Wald ratio	1	-0.068	0.031	2.84E-02	*PPARG*
**Spinal_cord_cervical**							
	rs116527582	Wald ratio	1	0.114	0.042	6.18E-03	*PPARG*
**Curcumin**							
**Hypothalamus**							
	rs530425	Wald ratio	1	-0.068	0.031	2.84E-02	*PPARG*
**Spinal_cord_cervical**							
	rs116527582	Wald ratio	1	0.114	0.042	6.18E-03	*PPARG*
NAC, N-Acetylcysteine							
ROSMAP, Religious Orders Study and Rush Memory and Aging Project							

Table S5. Two-sample MR of the targets on major depressive disorder.							
	SNPs	Test	SNP_number	Beta	Se	*P_value*	Genes
**NAC**							
**Amygdala**							
	rs11644783	Wald ratio	1	0.057	0.021	6.42E-03	*GRIN2A*
	rs3751972	Wald ratio	1	-0.017	0.007	1.52E-02	*NOS2*
**Cerebellar_Hemisphere**							
	rs75142508	Wald ratio	1	0.046	0.020	2.03E-02	*GRIN2A*
**Cerebellum**							
	rs6058209	Wald ratio	1	-0.048	0.019	1.40E-02	*GSS*
	rs1463132	Wald ratio	1	0.047	0.017	6.32E-03	*GRIN2A*
**Frontal_Cortex**							
	rs12780212	Wald ratio	1	-0.042	0.018	2.17E-02	*CHUK*
**Metformin**							
**Amygdala**							
	rs3210749	Wald ratio	1	-0.023	0.011	2.75E-02	*ETFDH*
**ubiquinone**							
**Amygdala**							
	rs2839606	Wald ratio	1	-0.035	0.015	1.64E-02	*NDUFV3*
**Anterior_cingulate_cortex**							
	rs11203218	Wald ratio	1	-0.035	0.015	2.25E-02	*NDUFV3*
**Hippocampus**							
	rs35893787	Wald ratio	1	-0.025	0.012	3.62E-02	*NDUFV3*
**Putamen_basal_ganglia**							
	rs2839606	Wald ratio	1	-0.030	0.012	1.64E-02	*NDUFV3*
**Resveratrol**							
**Anterior_cingulate_cortex**							
	rs4790213	Wald ratio	1	0.014	0.007	3.34E-02	*ALOX15*
	rs4542199	Wald ratio	1	0.033	0.011	3.60E-03	*NR1I3*
**Caudate_basal_ganglia**							
	rs9371563	Wald ratio	1	0.050	0.019	7.66E-03	*ESR1*
**Cerebellar_Hemisphere**							
	rs9906642	Wald ratio	1	0.015	0.007	2.35E-02	*ALOX15*
**Frontal_Cortex**							
	rs144547994	Wald ratio	1	-0.028	0.012	1.87E-02	*AHR*
	rs16876611	Wald ratio	1	0.073	0.027	8.03E-03	*PI4K2B*
**Hippocampus**							
	rs139727076	Wald ratio	1	0.035	0.016	3.08E-02	*PTGS2*
**Hypothalamus**							
	rs3732359	Wald ratio	1	-0.072	0.022	8.78E-04	*NR1I2*
**Nucleus_accumbens**							
	rs17441991	Wald ratio	1	-0.057	0.029	4.80E-02	*ALOX5*
**Spinal_cord_cervical**							
	rs4371852	Wald ratio	1	0.026	0.012	2.38E-02	*ESR1*
**ROSMAP**							
	rs1811442	Wald ratio	1	0.402	0.181	2.60E-02	*SNCA*
**Ginkgo biloba extract**							
**Caudate_basal_ganglia**							
	rs3751972	Wald ratio	1	0.023	0.009	1.52E-02	*NOS2*
**Cerebellar_Hemisphere**							
	rs3751972	Wald ratio	1	0.024	0.010	1.52E-02	*NOS2*
**Cerebellum**							
	rs3751972	Wald ratio	1	0.018	0.007	1.52E-02	*NOS2*
**Hippocampus**							
	rs11657699	Wald ratio	1	-0.019	0.007	1.01E-02	*NOS2*
**Hypothalamus**							
	rs2002846	Wald ratio	1	-0.032	0.010	1.58E-03	*PLA2G2A*
	rs3751972	Wald ratio	1	-0.014	0.006	1.52E-02	*NOS2*
**Nucleus_accumbens**							
	rs3751972	Wald ratio	1	0.021	0.009	1.52E-02	*NOS2*
**Putamen_basal_ganglia**							
	rs3751972	Wald ratio	1	0.022	0.009	1.52E-02	*NOS2*
**Spinal_cord_cervical**							
	rs3751972	Wald ratio	1	0.022	0.009	1.52E-02	*NOS2*
**Substantia_nigra**							
	rs3751972	Wald ratio	1	-0.013	0.005	1.52E-02	*NOS2*
**ROSMAP**							
	rs4921420	Wald ratio	1	0.931	0.317	3.37E-03	*GABRA1*
**Vitamin E**							
**Caudate_basal_ganglia**							
	rs4955416	Wald ratio	1	0.072	0.028	1.08E-02	*P4HTM*
**Cerebellum**							
	rs35289401	Wald ratio	1	0.048	0.022	3.11E-02	*SEC14L2*
	rs7007908	Wald ratio	1	0.047	0.020	1.88E-02	*PPP2CB*
**Hypothalamus**							
	rs3732359	Wald ratio	1	-0.072	0.022	8.78E-04	*NR1I2*
	rs28483846	Wald ratio	1	0.072	0.031	1.88E-02	*PPP2CB*
**Nucleus_accumbens**							
	rs17441991	Wald ratio	1	-0.057	0.029	4.80E-02	*ALOX5*
**Spinal_cord_cervical**							
	rs228882	Wald ratio	1	0.033	0.012	4.89E-03	*PRKCA*
**Melatonin**							
**Caudate_basal_ganglia**							
	rs9371563	Wald ratio	1	0.050	0.019	7.66E-03	*ESR1*
**Hypothalamus**							
	rs1862260	Wald ratio	1	-0.043	0.020	2.88E-02	*CALR*
**Spinal_cord_cervical**							
	rs4371852	Wald ratio	1	0.026	0.012	2.38E-02	*ESR1*
**Vitamin C**							
**Cerebellar_Hemisphere**							
	rs4384984	Wald ratio	1	0.088	0.018	1.98E-06	*P4HTM*
**Cerebellum**							
	rs6769821	Wald ratio	1	0.069	0.014	4.64E-07	*P4HTM*
**Cortex**							
	rs4955411	Wald ratio	1	0.078	0.028	4.70E-03	*P4HTM*
**Frontal_Cortex**							
	rs13064784	Wald ratio	1	0.046	0.023	4.80E-02	*P4HTM*
	rs28768122	Wald ratio	1	0.046	0.016	4.47E-03	*OGFOD2*
**Hippocampus**							
	rs4955416	Wald ratio	1	0.093	0.036	1.08E-02	*P4HTM*
**Hypothalamus**							
	rs6446205	Wald ratio	1	0.047	0.024	4.80E-02	*P4HTM*
	rs10864725	Wald ratio	1	0.048	0.023	3.43E-02	*EGLN1*
**Nucleus_accumbens**							
	rs143658733	Wald ratio	1	0.042	0.014	2.91E-03	*DBH*
**Putamen_basal_ganglia**							
	rs7373072	Wald ratio	1	0.069	0.027	1.08E-02	*P4HTM*
**ROSMAP**							
	rs7106917	Wald ratio	1	-0.109	0.036	2.24E-03	*BBOX1*
	rs4955426	Wald ratio	1	-0.306	0.116	8.42E-03	*P4HTM*
**Vitamin A**							
**Cerebellum**							
	rs12148849	Wald ratio	1	-0.034	0.012	4.11E-03	*ALDH1A3*
**Frontal_Cortex**							
	rs150543773	Wald ratio	1	-0.043	0.015	3.96E-03	*RDH11*
**Curcumin**							
**Cortex**							
	rs762803	Wald ratio	1	-0.030	0.014	3.43E-02	*GSTP1*
**Hypothalamus**							
	rs7945927	Wald ratio	1	-0.021	0.009	2.56E-02	*GSTP1*
**Nucleus_accumbens**							
	rs7945927	Wald ratio	1	-0.046	0.021	2.56E-02	*GSTP1*
**Substantia_nigra**							
	rs67296566	Wald ratio	1	0.033	0.017	4.71E-02	*ABCC5*
**ROSMAP**							
	rs7621975	Wald ratio	1	-0.410	0.166	1.37E-02	*ABCC5*
	rs7941648	Wald ratio	1	0.101	0.047	3.08E-02	*GSTP1*
NAC, N-Acetylcysteine							
ROSMAP, Religious Orders Study and Rush Memory and Aging Project							

Table S6. Two-sample MR of the targets on attention-deficit/hyperactivity disorder.							
	**SNPs**	**Test**	**SNP_number**	**Beta**	**Se**	***P_value***	**Genes**
**Vitamin C**							
**Amygdala**							
	rs6442126	Wald ratio	1	0.129	0.064	4.48E-02	*P4HTM*
**Anterior_cingulate_cortex**							
	rs10865952	Wald ratio	1	0.224	0.103	2.93E-02	*P4HTM*
**Cerebellar_Hemisphere**							
	rs4384984	Wald ratio	1	0.156	0.056	5.57E-03	*P4HTM*
	rs4069668	Wald ratio	1	-0.165	0.079	3.71E-02	*OGFOD2*
**Cerebellum**							
	rs6769821	Wald ratio	1	0.119	0.042	4.24E-03	*P4HTM*
**Cortex**							
	rs4955411	Wald ratio	1	0.207	0.083	1.25E-02	*P4HTM*
**Frontal_Cortex**							
	rs13064784	Wald ratio	1	0.209	0.071	3.49E-03	*P4HTM*
**Hypothalamus**							
	rs6446205	Wald ratio	1	0.214	0.073	3.57E-03	*P4HTM*
**Nucleus_accumbens**							
	rs323717	Wald ratio	1	0.185	0.069	7.29E-03	*P3H1*
**Spinal_cord_cervical**							
	rs6791234	Wald ratio	1	0.089	0.031	3.74E-03	*P4HTM*
**ROSMAP**							
	rs4379383	Wald ratio	1	0.805	0.383	3.55E-02	*PLOD3*
	rs4955426	Wald ratio	1	-0.921	0.353	9.12E-03	*P4HTM*
**NAC**							
**Caudate_basal_ganglia**							
	rs12932206	Wald ratio	1	-0.115	0.051	2.37E-02	*GRIN2A*
**Frontal_Cortex**							
	rs7657780	Wald ratio	1	0.254	0.111	2.15E-02	*SLC7A11*
	rs76300578	Wald ratio	1	0.128	0.061	3.75E-02	*NFKB1*
**Hippocampus**							
	rs12932206	Wald ratio	1	-0.173	0.076	2.37E-02	*GRIN2A*
**Putamen_basal_ganglia**							
	rs12932206	Wald ratio	1	-0.083	0.037	2.37E-02	*GRIN2A*
**Spinal_cord_cervical**							
	rs12932206	Wald ratio	1	-0.048	0.021	2.37E-02	*GRIN2A*
**ROSMAP**							
	rs2236271	Wald ratio	1	0.494	0.243	4.24E-02	*GSS*
	rs220593, rs10772724	Inverse variance weighted	2	-1.019	0.494	3.91E-02	*GRIN2B*
**Resveratrol**							
**Caudate_basal_ganglia**							
	rs9371563	Wald ratio	1	0.153	0.059	9.64E-03	*ESR1*
**Cortex**							
	rs75756429	Wald ratio	1	0.110	0.049	2.43E-02	*ESR1*
**Frontal_Cortex**							
	rs76369590	Wald ratio	1	0.205	0.083	1.36E-02	*ITGA5*
**Spinal_cord_cervical**							
	rs4371852	Wald ratio	1	0.094	0.036	9.60E-03	*ESR1*
**Melatonin**							
**Caudate_basal_ganglia**							
**13980**	rs9371563	Wald ratio	1	0.153	0.059	9.64E-03	*ESR1*
**Cortex**							
	rs75756429	Wald ratio	1	0.110	0.049	2.43E-02	*ESR1*
**Spinal_cord_cervical**							
	rs4371852	Wald ratio	1	0.094	0.036	9.60E-03	*ESR1*
**Substantia_nigra**							
	rs302864	Wald ratio	1	0.074	0.033	2.44E-02	*EPX*
**Vitamin E**							
**Cerebellum**							
**14077**	rs77290814	Wald ratio	1	-0.296	0.146	4.21E-02	*PRKCA*
**Vitamin D**							
**Spinal_cord_cervical**							
	rs886441	Wald ratio	1	-0.087	0.041	3.32E-02	*VDR*
**Curcumin**							
**Spinal_cord_cervical**							
	rs886441	Wald ratio	1	-0.087	0.041	3.32E-02	*VDR*
NAC, N-Acetylcysteine							
ROSMAP, Religious Orders Study and Rush Memory and Aging Project							

Table S7. Two-sample MR of the targets on autism spectrum disorder.							
	**SNPs**	**Test**	**SNP_number**	**Beta**	**Se**	***P_value***	**Genes**
**NAC**							
**Amygdala**							
	rs11644783	Wald ratio	1	0.187	0.069	6.88E-03	*GRIN2A*
**Cerebellar_Hemisphere**							
	rs12003467	Wald ratio	1	0.084	0.033	1.17E-02	*GRIN3A*
**Substantia_nigra**							
	rs57283800	Wald ratio	1	-0.288	0.114	1.13E-02	*GRIN1*
**Metformin**							
**Anterior_cingulate_cortex**							
	rs72693373	Wald ratio	1	0.082	0.040	4.14E-02	*ETFDH*
**Frontal_Cortex**							
	rs10774515	Wald ratio	1	-0.121	0.053	2.20E-02	*PRKAB1*
**Hypothalamus**							
	rs11100191	Wald ratio	1	0.065	0.031	3.70E-02	*ETFDH*
**Nucleus_accumbens**							
	rs67442921	Wald ratio	1	0.082	0.040	3.94E-02	*ETFDH*
**Substantia_nigra**							
	rs55822924	Wald ratio	1	0.068	0.032	3.34E-02	*ETFDH*
**Ginkgo biloba extract**							
**Caudate_basal_ganglia**							
	rs7706918	Wald ratio	1	-0.287	0.145	4.75E-02	*GABRA1*
**Spinal_cord_cervical**							
	rs114315666	Wald ratio	1	0.092	0.046	4.88E-02	*GABRA1*
**ROSMAP**							
	rs4921381	Wald ratio	1	-2.105	0.715	3.26E-03	*GABRB2*
	rs4921420	Wald ratio	1	2.114	1.017	3.77E-02	*GABRA1*
**Vitamin A**							
**ROSMAP**							
	rs1342022	Wald ratio	1	-0.790	0.377	3.63E-02	*ALDH1A1*
**Vitamin E**							
**Caudate_basal_ganglia**							
	rs5994305	Wald ratio	1	0.135	0.067	4.21E-02	*SEC14L2*
**Cerebellar_Hemisphere**							
	rs5753113	Wald ratio	1	0.169	0.062	6.67E-03	*SEC14L2*
**Frontal_Cortex**							
	rs4791035	Wald ratio	1	0.104	0.050	3.71E-02	*PRKCA*
**Nucleus_accumbens**							
	rs5994306	Wald ratio	1	0.145	0.073	4.61E-02	*SEC14L2*
**Melatonin**							
**Cerebellum**							
	rs514209	Wald ratio	1	-0.146	0.064	2.27E-02	*RORB*
**Cortex**							
	rs75756429	Wald ratio	1	0.107	0.050	3.28E-02	*ESR1*
**Substantia_nigra**							
	rs302864	Wald ratio	1	0.070	0.033	3.13E-02	*EPX*
**Resveratrol**							
**Cortex**							
	rs75756429	Wald ratio	1	0.107	0.050	3.28E-02	*ESR1*
**Frontal_Cortex**							
	rs16876611	Wald ratio	1	0.180	0.085	3.37E-02	*PI4K2B*
**Hippocampus**							
	rs1005696	Wald ratio	1	-0.139	0.069	4.40E-02	*CBR1*
**Hypothalamus**							
	rs530425	Wald ratio	1	0.141	0.048	3.15E-03	*PPARG*
**ROSMAP**							
	rs2835267	Wald ratio	1	0.316	0.155	4.11E-02	*CBR1*
	rs1811442	Wald ratio	1	1.322	0.607	2.93E-02	*SNCA*
**Ubiquinone**							
**Hippocampus**							
	rs62345236	Wald ratio	1	0.234	0.090	9.11E-03	*SDHA*
**Nucleus_accumbens**							
	rs55671190	Wald ratio	1	0.171	0.066	9.20E-03	*SDHA*
**Spinal_cord_cervical**							
	rs62329990	Wald ratio	1	0.111	0.037	2.70E-03	*SDHA*
**Vitamin C**							
**Hippocampus**							
	rs1416912	Wald ratio	1	-0.140	0.059	1.81E-02	*EGLN1*
**Nucleus_accumbens**							
	rs2739514	Wald ratio	1	-0.142	0.069	3.92E-02	*EGLN1*
**ROSMAP**							
	rs6541266	Wald ratio	1	1.652	0.663	1.27E-02	*EGLN1*
**Curcumin**							
**Hippocampus**							
	rs1005696	Wald ratio	1	-0.139	0.069	4.40E-02	*CBR1*
**Hypothalamus**							
	rs530425	Wald ratio	1	0.141	0.048	3.15E-03	*PPARG*
**Putamen_basal_ganglia**							
	rs1554395	Wald ratio	1	-0.158	0.071	2.58E-02	*ABCC5*
**ROSMAP**							
	rs2835267	Wald ratio	1	0.316	0.155	4.11E-02	*CBR1*
**Omega-3**							
**Hypothalamus**							
	rs530425	Wald ratio	1	0.141	0.048	3.15E-03	*PPARG*
NAC, N-Acetylcysteine							
ROSMAP, Religious Orders Study and Rush Memory and Aging Project							

Table S8. Two-sample MR of the targets on obsessive-compulsive disorder.

	SNPs	Test	SNP_number	Beta	Se	*P_value*	Genes
**Vitamin C**							
**Amygdala**							
	rs1375583	Wald ratio	1	-0.257	0.119	3.09E-02	*PLOD2*
**Caudate_basal_ganglia**							
	rs13088158	Wald ratio	1	-0.388	0.160	1.52E-02	*PLOD2*
**Cerebellar_Hemisphere**							
	rs4660653	Wald ratio	1	-0.466	0.190	1.42E-02	*P3H1*
	rs7103602	Wald ratio	1	0.299	0.151	4.78E-02	*ALKBH3*
**Frontal_Cortex**							
	rs45525835	Wald ratio	1	0.688	0.273	1.18E-02	*PLOD1*
	rs147289644	Wald ratio	1	-0.599	0.297	4.40E-02	*BBOX1*
**Hypothalamus**							
	rs727838	Wald ratio	1	-0.386	0.187	3.89E-02	*BBOX1*
**Omega-3**							
**Anterior_cingulate_cortex**							
	rs12600694	Wald ratio	1	-0.475	0.204	1.97E-02	*SREBF1*
**NAC**							
**Caudate_basal_ganglia**							
	rs72640377	Wald ratio	1	0.500	0.232	3.14E-02	*IKBKB*
**Substantia_nigra**							
	rs146318115	Wald ratio	1	-0.298	0.122	1.46E-02	*GRIN2D*
**Cerebellum**							
	rs1463132	Wald ratio	1	-0.316	0.137	2.10E-02	*GRIN2A*
**Frontal_Cortex**							
	rs76300578	Wald ratio	1	0.334	0.153	2.89E-02	*NFKB1*
**Hypothalamus**							
	rs7198417	Wald ratio	1	0.217	0.100	2.93E-02	*GRIN2A*
**Ubiquinone**							
**Putamen_basal_ganglia**							
	rs55671190	Wald ratio	1	-0.325	0.156	3.70E-02	*SDHA*
**Metformin**							
**Caudate_basal_ganglia**							
	rs1978108	Wald ratio	1	-0.405	0.150	6.84E-03	*PRKAB1*
**Cerebellum**							
	rs7310266	Wald ratio	1	-0.435	0.163	7.60E-03	*PRKAB1*
**Putamen_basal_ganglia**							
	rs7304996	Wald ratio	1	-0.343	0.129	7.60E-03	*PRKAB1*
**ROSMAP**							
	rs278155	Wald ratio	1	-5.947	1.108	4.47E-03	*PRKAB1*
**Resveratrol**							
**Caudate_basal_ganglia**							
	rs17552473	Wald ratio	1	-0.625	0.268	1.94E-02	*MTNR1B*
**Cortex**							
	rs13198902	Wald ratio	1	-0.232	0.097	1.67E-02	*NQO2*
**Hypothalamus**							
	rs3732359	Wald ratio	1	-0.391	0.169	2.06E-02	*NR1I2*
**Melatonin**							
**Caudate_basal_ganglia**							
	rs17552473	Wald ratio	1	-0.625	0.268	1.94E-02	*MTNR1B*
**Cortex**							
	rs13198902	Wald ratio	1	-0.232	0.097	1.67E-02	*NQO2*
**Spinal_cord_cervical**							
	rs62082141	Wald ratio	1	-0.167	0.077	2.93E-02	*MPO*
**Vitamin A**							
**Cerebellar_Hemisphere**							
	rs7146599	Wald ratio	1	0.253	0.099	1.04E-02	*DHRS4*
**Cerebellum**							
	rs7146599	Wald ratio	1	0.205	0.080	1.04E-02	*DHRS4*
**Frontal_Cortex**							
	rs150543773	Wald ratio	1	-0.297	0.117	1.11E-02	*RDH11*
	rs11638367	Wald ratio	1	-0.301	0.104	3.81E-03	*ALDH1A3*
**Spinal_cord_cervical**							
	rs6759239	Wald ratio	1	-0.335	0.154	2.95E-02	*RETSAT*
**Ginkgo biloba extract**							
**Cortex**							
	rs58977775	Wald ratio	1	-0.642	0.302	3.36E-02	*GABRA1*
**Spinal_cord_cervical**							
	rs606980	Wald ratio	1	-0.129	0.063	4.10E-02	*PLA2G2A*
**Vitamin E**							
**Hippocampus**							
	rs42937	Wald ratio	1	-0.287	0.143	4.52E-02	*SEC14L2*
**Hypothalamus**							
	rs3732359	Wald ratio	1	-0.391	0.169	2.06E-02	*NR1I2*
**ROSMAP**							
	rs2240422, rs11704395	Inverse variance weighted	2	1.232	1.710	4.51E-02	*SEC14L2*

NAC, N-Acetylcysteine

ROSMAP, Religious Orders Study and Rush Memory and Aging Project

Table S9. Two-sample MR of the targets on anorexia nervosa.							
	**SNPs**	**Test**	**SNP_number**	**Beta**	**Se**	***P_value***	**Genes**
**Resveratrol**							
**Amygdala**							
	rs77810888	Wald ratio	1	0.189	0.094	4.37E-02	*AHR*
**Ubiquinone**							
**Cerebellar_Hemisphere**							
	rs71320572	Wald ratio	1	-0.122	0.051	1.66E-02	*NDUFV3*
**Cerebellum**							
	rs71320572	Wald ratio	1	-0.114	0.047	1.66E-02	*NDUFV3*
**Cortex**							
	rs4148974	Wald ratio	1	0.220	0.098	2.43E-02	*NDUFV3*
**Hypothalamus**							
	rs4148974	Wald ratio	1	0.289	0.128	2.43E-02	*NDUFV3*
**Nucleus_accumbens**							
	rs4148974	Wald ratio	1	0.239	0.106	2.43E-02	*NDUFV3*
**Vitamin C**							
**Cerebellum**							
	rs8016136	Wald ratio	1	0.230	0.115	4.58E-02	*EGLN3*
**Frontal_Cortex**							
	rs75543548	Wald ratio	1	0.254	0.122	3.76E-02	*PHYH*
**Nucleus_accumbens**							
	rs143658733	Wald ratio	1	0.242	0.090	7.31E-03	*DBH*
	rs138098518	Wald ratio	1	-0.236	0.119	4.76E-02	*OGFOD2*
**Vitamin A**							
**Cortex**							
	rs12911338	Wald ratio	1	-0.158	0.076	3.67E-02	*ALDH1A3*
**Vitamin D**							
**ROSMAP**							
	rs222047	Wald ratio	1	-0.600	0.305	4.97E-02	*GC*
NAC, N-Acetylcysteine							
ROSMAP, Religious Orders Study and Rush Memory and Aging Project							

**Table S10. Mediation analysis.**												
Target	Mediator	beta1	se1	p1	method	Disease	beta2	se2	p2	method	beta0	proportion
OGFOD2_Cerebellum	Vitamin c	0.018	0.030	5.37E-01	Wald ratio	SCZ	0.070	0.108	5.20E-01	Inverse variance weighted	-0.244	-0.005
**OGFOD2_Cerebellum**	**Fluid intelligence score**	**0.179**	**0.041**	**1.48E-05**	**Wald ratio**	**SCZ**	**0.170**	**0.029**	**5.20E-09**	**Inverse variance weighted**	**-0.244**	**-0.111**
OGFOD2_Cerebellum	Duration to complete numeric path	0.028	0.024	2.48E-01	Wald ratio	SCZ	-0.306	0.154	4.62E-02	Inverse variance weighted	-0.244	0.035
OGFOD2_Cerebellum	Duration to complete alphanumeric path	0.013	0.024	5.96E-01	Wald ratio	SCZ	-0.624	0.119	1.52E-07	Inverse variance weighted	-0.244	0.033
**OGFOD2_Cerebellum**	**Number of incorrect matches in round**	**-0.025**	**0.011**	**2.29E-02**	**Wald ratio**	**SCZ**	**-0.906**	**0.149**	**1.17E-09**	**Inverse variance weighted**	**-0.244**	**-0.086**
OGFOD2_Cerebellum	Number of correct matches in round	0.002	0.007	7.47E-01	Wald ratio	SCZ	-0.072	0.406	8.58E-01	Inverse variance weighted	-0.244	0.001
OGFOD2_Cerebellum	Maximum digits remembered correctly	0.070	0.052	1.76E-01	Wald ratio	SCZ	-0.048	0.074	5.17E-01	Inverse variance weighted	-0.244	0.014
OGFOD2_Cerebellum	Prospective memory result	-0.017	0.010	1.04E-01	Wald ratio	SCZ	-0.484	0.262	6.50E-02	Inverse variance weighted	-0.244	-0.032
RORB_Cerebellum	Fluid intelligence score	0.002	0.035	9.52E-01	Wald ratio	SCZ	0.170	0.029	5.20E-09	Inverse variance weighted	0.234	0.002
**RORB_Cerebellum**	**Duration to complete numeric path**	**-0.058**	**0.021**	**5.47E-03**	**Wald ratio**	**SCZ**	**-0.306**	**0.154**	**4.62E-02**	**Inverse variance weighted**	**0.234**	**0.076**
**RORB_Cerebellum**	**Duration to complete alphanumeric path**	**-0.045**	**0.021**	**2.97E-02**	**Wald ratio**	**SCZ**	**-0.624**	**0.119**	**1.52E-07**	**Inverse variance weighted**	**0.234**	**0.120**
RORB_Cerebellum	Number of incorrect matches in round	-0.009	0.009	3.54E-01	Wald ratio	SCZ	-0.906	0.149	1.17E-09	Inverse variance weighted	0.234	0.034
RORB_Cerebellum	Number of correct matches in round	-0.004	0.006	5.10E-01	Wald ratio	SCZ	-0.072	0.406	8.58E-01	Inverse variance weighted	0.234	0.001
RORB_Cerebellum	Maximum digits remembered correctly	0.000	0.030	9.87E-01	Wald ratio	SCZ	-0.048	0.074	5.17E-01	Inverse variance weighted	0.234	0.000
RORB_Cerebellum	Prospective memory result	0.012	0.009	1.89E-01	Wald ratio	SCZ	-0.484	0.262	6.50E-02	Inverse variance weighted	0.234	-0.023
PRKCB_ROSMAP	Vitamin e	0.127	0.171	4.57E-01	Inverse vari	BD	0.007	0.081	9.27E-01	Inverse variance weighted	-1.448	-0.001
PRKCB_ROSMAP	Fluid intelligence score	-0.174	0.335	6.05E-01	Inverse vari	BD	0.035	0.024	1.49E-01	Inverse variance weighted	-1.448	0.004
PRKCB_ROSMAP	Duration to complete numeric path	-0.040	0.140	7.73E-01	Inverse vari	BD	-0.142	0.105	1.78E-01	Inverse variance weighted	-1.448	-0.004
PRKCB_ROSMAP	Duration to complete alphanumeric path	0.002	0.139	9.88E-01	Inverse vari	BD	-0.288	0.086	8.03E-04	Inverse variance weighted	-1.448	0.000
PRKCB_ROSMAP	Number of incorrect matches in round	-0.020	0.163	9.03E-01	Inverse vari	BD	-0.548	0.116	2.21E-06	Inverse variance weighted	-1.448	-0.007
PRKCB_ROSMAP	Number of correct matches in round	-0.015	0.040	7.03E-01	Inverse vari	BD	0.187	0.295	5.26E-01	Inverse variance weighted	-1.448	0.002
PRKCB_ROSMAP	Maximum digits remembered correctly	0.247	0.216	2.53E-01	Inverse vari	BD	0.005	0.060	9.40E-01	Inverse variance weighted	-1.448	-0.001
PRKCB_ROSMAP	Prospective memory result	0.014	0.059	8.11E-01	Inverse vari	BD	-0.309	0.185	9.45E-02	Inverse variance weighted	-1.448	0.003
P4HTM_Cerebellum	Vitamin c	0.000	0.017	9.78E-01	Wald ratio	MDD	-0.039	0.050	4.33E-01	Inverse variance weighted	0.069	0.000
**P4HTM_Cerebellum**	**Fluid intelligence score**	**0.050**	**0.024**	**3.70E-02**	**Wald ratio**	**MDD**	**-0.029**	**0.011**	**7.01E-03**	**Inverse variance weighted**	**0.069**	**-0.021**
**P4HTM_Cerebellum**	**Duration to complete numeric path**	**-0.036**	**0.014**	**1.00E-02**	**Wald ratio**	**MDD**	**0.109**	**0.053**	**4.10E-02**	**Inverse variance weighted**	**0.069**	**-0.054**
**P4HTM_Cerebellum**	**Duration to complete alphanumeric path**	**-0.053**	**0.014**	**1.53E-04**	**Wald ratio**	**MDD**	**0.110**	**0.042**	**8.07E-03**	**Inverse variance weighted**	**0.069**	**-0.079**
**P4HTM_Cerebellum**	**Number of incorrect matches in round**	**-0.018**	**0.006**	**6.20E-03**	**Wald ratio**	**MDD**	**0.219**	**0.053**	**3.28E-05**	**Inverse variance weighted**	**0.069**	**-0.053**
P4HTM_Cerebellum	Number of correct matches in round	0.008	0.004	4.97E-02	Wald ratio	MDD	-0.267	0.158	9.07E-02	Inverse variance weighted	0.069	-0.030
P4HTM_Cerebellum	Maximum digits remembered correctly	0.004	0.020	8.46E-01	Wald ratio	MDD	0.015	0.031	6.31E-01	Inverse variance weighted	0.069	0.001
P4HTM_Cerebellum	Prospective memory result	-0.001	0.006	8.69E-01	Wald ratio	MDD	0.073	0.076	3.33E-01	Inverse variance weighted	0.069	-0.001
P4HTM_Cerebellar_H	Vitamin c	0.003	0.023	9.08E-01	Wald ratio	MDD	-0.039	0.050	4.33E-01	Inverse variance weighted	0.088	-0.001
**P4HTM_Cerebellar_H**	**Fluid intelligence score**	**0.072**	**0.032**	**2.46E-02**	**Wald ratio**	**MDD**	**-0.029**	**0.011**	**7.01E-03**	**Inverse variance weighted**	**0.088**	**-0.024**
**P4HTM_Cerebellar_H**	**Duration to complete numeric path**	**-0.051**	**0.019**	**7.24E-03**	**Wald ratio**	**MDD**	**0.109**	**0.053**	**4.10E-02**	**Inverse variance weighted**	**0.088**	**-0.060**
**P4HTM_Cerebellar_H**	**Duration to complete alphanumeric path**	**-0.075**	**0.019**	**8.59E-05**	**Wald ratio**	**MDD**	**0.110**	**0.042**	**8.07E-03**	**Inverse variance weighted**	**0.088**	**-0.086**
**P4HTM_Cerebellar_H**	**Number of incorrect matches in round**	**-0.024**	**0.009**	**5.94E-03**	**Wald ratio**	**MDD**	**0.219**	**0.053**	**3.28E-05**	**Inverse variance weighted**	**0.088**	**-0.056**
P4HTM_Cerebellar_H	Number of correct matches in round	0.012	0.005	3.42E-02	Wald ratio	MDD	-0.267	0.158	9.07E-02	Inverse variance weighted	0.088	-0.034
P4HTM_Cerebellar_H	Maximum digits remembered correctly	0.004	0.027	8.82E-01	Wald ratio	MDD	0.015	0.031	6.31E-01	Inverse variance weighted	0.088	0.001
P4HTM_Cerebellar_H	Prospective memory result	-0.004	0.008	6.00E-01	Wald ratio	MDD	0.073	0.076	3.33E-01	Inverse variance weighted	0.088	-0.003
ROSMAP, Religious Orders Study and Rush Memory and Aging Project; SCZ, schiophrenia; BD, bipolar disorder; MDD, major depressive disorder.												
